# Development of *Capsicum* EST–SSR markers for species identification and *in silico* mapping onto the tomato genome sequence

**DOI:** 10.1007/s11032-012-9774-z

**Published:** 2012-08-11

**Authors:** Kenta Shirasawa, Kohei Ishii, Cholgwang Kim, Tomohiro Ban, Munenori Suzuki, Takashi Ito, Toshiya Muranaka, Megumi Kobayashi, Noriko Nagata, Sachiko Isobe, Satoshi Tabata

**Affiliations:** 1 Kazusa DNA Research Institute, 2-6-7 Kazusa-Kamatari, Kisarazu, Chiba, 291-0818 Japan; 2 Kihara Institute for Biological Research, Yokohama City University, Yokohama, Kanagawa, 244-0813 Japan; 3 Osaka University, Suita, Osaka, 565-0871 Japan; 4 Japan Women’s University, Bunkyo, Tokyo, 112-8681 Japan

**Keywords:** *Capsicum* spp., DNA barcoding, EST–SSR marker, Species identification, Solanaceae

## Abstract

*Capsicum* spp. are widely cultivated for use as vegetables and spices. The Kihara Institute for Biological Research, Yokohama City University, Japan, has stocks of approximately 800 lines of *Capsicum* spp. collected from various regions of Central and South America, the regions of origin for *Capsicum* spp. In this study, 5,751 primer pairs for simple sequence repeat markers, based on 118,060 publicly available sequences of expressed sequence tags of *Capsicum annuum*, were designed and subjected to a similarity search against the genomic sequence of tomato, a model Solanaceae species. Nucleotide sequences spanning 2,245 *C. annuum* markers were successfully mapped onto the tomato genome, and 96 of these, which spanned the entire tomato genome, were selected for further analysis. In genotyping analysis, 60 out of the 77 markers that produced specific DNA amplicons showed polymorphism among the *Capsicum* lines examined. On the basis of the resulting data, the 192 tested lines were grouped into five main clusters. The additional sequencing analysis of the plastid genes, *matK* and *rbcL*, divided the resources into three groups. As a result, 19 marker loci exhibited genotypes specific to species and cluster, suggesting that the DNA markers are useful for species identification. Information on the DNA markers will contribute to *Capsicum* genetics, genomics, and breeding.

**Electronic supplementary material:**

The online version of this article (doi:10.1007/s11032-012-9774-z) contains supplementary material, which is available to authorized users.

## Introduction

The genus *Capsicum* is a member of the family Solanaceae. The Solanaceae includes the genus *Solanum*, to which tomato (*Solanum lycopersicum*) and potato (*S. tuberosum*) also belong. The genus *Capsicum* includes several species of importance as food and spice crops. In addition, extracts are used as components of color dyes and medications. This genus includes several cultivated peppers, e.g., *Capsicum annuum,* including bell pepper, jalapeno, New Mexico chile, ancho, Anaheim chile, and banana pepper; *Capsicum baccatum*, including Ají amarillo; *Capsicum chinense,* including habanero; *Capsicum frutescens,* including Tabasco; and *Capsicum pubescens*, including rocoto peppers (Paran et al. 2007). All of these have interspecific compatibility with each other except for *C. pubescens* (Walsh and Hoot 2001). While the complete genome sequences of both tomato and potato have been released (The Potato Genome Sequencing Consortium 2011; The Tomato Genome Consortium 2012), that of *Capsicum* has not been determined due to its large genome size (3.3 Gb, Moscone et al. 2003). However, other resources for genomic and genetic studies, viz., expressed sequence tag (EST) sequences, molecular markers, and genetic linkage maps, have been developed and used in quantitative trait loci (QTL) mapping studies, genetic diversity analyses, and comparative genomics in the genus *Capsicum* (Jung et al. 2010; Lee et al. 2004; Minamiyama et al. 2006; Paran et al. 2004; Wu et al. 2009; Yi et al. 2006; Miura et al. 2012). Such efforts have revealed that the pepper genome has significant synteny with the tomato genome (Wu et al. 2009).

The conservation of divergent plants is important from the points of views of biology, ecology, and breeding. Therefore, seeds have been stocked as genetic resources in several genetic resource centers and gene banks, e.g., the National BioResource Project (Kurata et al. 2010) and the Global Crop Diversity Trust (Swaminathan 2009). In such genetic resource centers, classification and identification of the genetic resources are important for the management of the stocks. The Kihara Institute for Biological Research (KIBR), Yokohama City University, Japan, is also a genetic resource center for *Capsicum* spp. and has kept approximately 800 lines collected from the center of origin of *Capsicum*, i.e., Central and South America. The species of the *Capsicum* stocks have been carefully classified according to the 12 criteria of the standardized phenotypic indexes of the International Plant Genetic Resource Institute, Asian Vegetable Research and Development Center, and Centro Agronómico Tropical de Investigación y Enseñanza of Costa Rica (IPGRI, AVRDC, and CATIE 1995). However, misidentification of species has sometimes occurred because phenotypic traits are often altered by environmental conditions. In addition, phenotypic classification using indexes requires skilled labor, time, and large fields in which to grow the plants. Consequently, this method is expensive and often impractical.

DNA sequence polymorphism is reliable, because it is not affected by environmental conditions. Furthermore, analysis of DNA polymorphism is a low-cost approach to the classification of species due to its requirements of fewer samples and less time and labor. The genetic diversity of the genus *Capsicum* has been investigated using DNA markers, mainly random amplified polymorphic DNA (RAPD) and amplified fragment length polymorphism (AFLP) markers (Oyama et al. 2006; Paran et al. 1998; Rodriguez et al. 1999). Such fingerprinting methods detect multi-locus polymorphism at the same time. Single nucleotide polymorphism (SNP) markers have also been used to identify *Capsicum* species (Jeong et al. 2010; Jung et al. 2010). SNP markers generally identify bi-allelic polymorphisms. The transferability of SNP markers to other species or lines is less than that of other marker systems. Therefore, for SNP analysis, large numbers of markers are generally required for diversity analysis. Simple sequence repeat (SSR), or microsatellite, markers detect differences in the lengths of mono- to hexa-nucleotide repeat sequences. SSR markers constitute a useful tool for genetic diversity analysis, in that they enable multi-allele detection, are highly transferable across species, and are flexible enough so that they can be used with various laboratory systems (Kalia et al. 2011). SSR markers can be classified into two categories: genomic SSRs and EST–SSRs, which are designed from whole-genome and mRNA transcript sequences, respectively (Kalia et al. 2011). EST–SSRs can be expected to have greater transferability between species/genera than genomic SSRs, since gene-coding regions are more likely to be conserved among related species/genera. In *Capsicum*, SSR markers developed from ESTs and SSR-enriched genomic libraries have been applied to the construction of linkage maps (Minamiyama et al. 2006; Yi et al. 2006). In addition, short and standardized DNA regions, i.e., “barcodes”, have been used as a tool for species identification (Hebert et al. 2003). In plants, the *matK* and *rbcL* loci in plastid DNA have been proposed as barcodes (CBLO Plant Working Group 2009).

To characterize the genetic diversity of the *Capsicum* lines stocked in the KIBR, we performed polymorphism analysis with EST–SSR markers and the plastid DNA barcode sequences. The primers for the EST–SSR markers were designed based on flanking regions of SSRs identified in publicly available ESTs of *C. annuum*. A BLAST search to the tomato genome was conducted using the ESTs from which these primers were designed (The Tomato Genome Consortium 2012). Based on this search, 96 EST–SSR markers, which spanned the entire tomato genome, were selected for the polymorphism analysis of *Capsicum* stocks. In addition, *matK* and *rbcL* barcode sequences from plastid DNA were also analyzed. The genetic diversity of the *Capsicum* spp. was therefore characterized by both EST–SSR marker-based analyses and sequencing of plastid DNA.

## Materials and methods

### Plant materials

A total of 186 samples of *Capsicum* genetic resources, consisting of 30 *C. annuum*, 21 *C. baccatum*, 85 *C. chinense*, 25 *C. frutescens*, one *C. pubescens*, and 24 *Capsicum* lines for which species were not identified, were selected from the active stocks of the KIBR. In addition, samples from five local Japanese landraces (*C. annuum*: Fushimi-Amanaga, Ougon, Shishi-Togarashi, and Takanotsume; and *C. frutescenes*: Okinawa-Togarashi) and one globally-cultivated line (*C. frutescenes*: Tabasco) were also used. The accession numbers and the countries of origin of the samples are listed in Supplementary Table S1.

### Development, similarity searches, and genotyping of EST–SSR markers

EST sequences of *C. annuum* were obtained from the NCBI database in April 2010 (http://www.ncbi.nlm.nih.gov). Primers for the EST–SSR markers were designed from the flanking sequences of di-, tri-, or tetra-nucleotide SSR motifs as described in our previous study (Koilkonda et al. 2012; Shirasawa et al. 2010; Shirasawa et al. 2011).

These EST sequences were subjected to a tBLASTx (Altschul et al. 1997) search of the tomato genome sequence SL2.30 (http://solgenomics.net), which was the latest version at the time of data analysis. These sequence similarities were judged to be significant when the E-value was <1e−50.

For each sample, genomic DNA was isolated from leaves using the DNeasy Plant mini prep kit (Qiagen). DNA concentration for each sample was determined using NanoDrop (Thermo Scientific). PCR and subsequent fluorescent fragment analysis were performed as described in Shirasawa et al. (2010). The expected heterozygosity (*HZ*) of each marker was calculated using the following formula:

$$ HZ = 1 - \sum\limits_{i = 1}^{n} {p_{i}^{2} } $$

where *p*_*i*_ is the frequency of the *i*th of *n* alleles.

### Sequencing of *matK* and *rbcL* genes

The universal primers (5′-CGTACAGTACTTTTGTGTTTACGAG-3′ and 5′-ACCCAGTCCATCTGGAAATCTTGGTTC-3′ for *matK*, and 5′-ATGTCACCACAAACAGAGACTAAAGC-3′ and 5′-GTAAAATCAAGTCCACCRCG-3′ for *rbcL*) were used to amplify DNA fragments from the chloroplast *matK* and *rbcL* genes (CBLO Plant Working Group 2009). PCR reactions were performed using 0.5 ng genomic DNA in each 5-μl reaction. In addition to template DNA, PCR reaction mixes contained 1× PCR buffer (Bioline, UK), 3 mM MgCl_2_, 0.04 U BIOTAQ DNA polymerase (Bioline, UK), 0.2 mM dNTPs, and 0.8 μM of each primer. The thermal cycling conditions were as follows: 1 min initial denaturation at 94 °C; 35 cycles of 30 s denaturation at 94 °C, 30 s annealing at 55 °C, and 1 min extension at 72 °C; and 3 min final extension at 72 °C. The amplified DNAs were treated with the ExoSAP-IT kit (GE Healthcare), which cleans up the reaction by dephosphorylating dNTPs and degrading primers that were not incorporated into the PCR products. These products were then used as templates for bidirectional sequencing analysis using the BigDye Terminator v3.1 Cycle Sequencing Kit (Applied Biosystems) and the DNA sequencer ABI 3730*xl* (Applied Biosystems).

### Clustering analysis based on the EST–SSR markers

The genetic distances and Jaccard’s similarity coefficients of all combinations of any two samples were calculated from the genotypic data using GGT2 software (van Berloo 2008). A dendrogram of the samples was established using the neighbor-joining method in MEGA5 software (Tamura et al. 2011).

## Results

### Features of SSRs from ESTs

A total of 118,060 EST sequences of *C. annuum* were obtained from the NCBI DNA database. After *in silico* data mining, 5,751 non-redundant EST–SSR markers were generated and designated as CaES (*C*. *a* *nnuum* EST–SSR) markers, out of which 75 were the same loci as reported by Yi et al. (2006) (Supplementary Table S2). Of the SSR motifs identified in the CaES markers, 4,311 (75.0 %) were trinucleotide repeats, 557 (9.7 %) were dinucleotide repeats, and 882 (15.3 %) were tetranucleotide repeats (Supplementary Fig. S1).

**Fig. 1:**
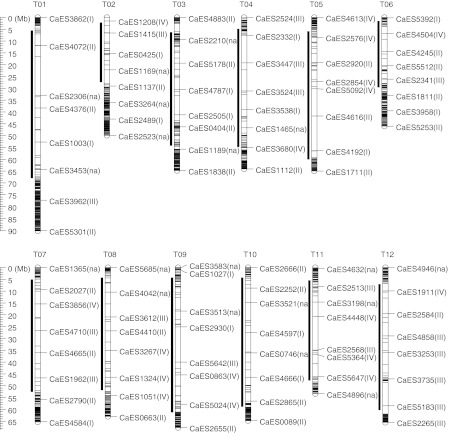
Map positions of the CaES markers on the tomato genome. The tomato chromosomes (T01–T12) are indicated in physical length. *Vertical bars* on the *left side* of the chromosomes show the heterochromatic regions. *Horizontal lines* on the chromosomes indicate the positions of the CaES markers; those analyzed in this study are shown with marker names. Descriptions in *parentheses* following the marker names indicate the marker types: *I* codominant polymorphic markers, *II* mixture of codominant and dominant polymorphic markers, *III* monomorphic markers, *IV* dominant polymorphic markers, *na* no amplification or multiple bands

The distributions of the EST–SSR markers on the tomato genome were investigated using BLAST. Of the 5,751 EST sequences from which the SSR primers were designed, 2,245 (39.0 %) showed significant similarity to the tomato genome sequences (SL2.30), while the positions of the mapped *C. annuum* ESTs on the tomato genome were highly biased (Fig. 1; Supplementary Table S2).

### Genotyping of the 192 lines using the 96 EST–SSR markers

Of the 2,245 mapped EST–SSR markers, 96 were selected for the diversity analysis of the 192 pepper samples to cover the tomato chromosome with constant intervals (eight markers per chromosome) (Fig. 1; Supplementary Table S2). Because 19 of the 96 selected markers gave no PCR amplicons and multiple bands, these markers were eliminated from the following analysis. The other 77 markers, which yielded one or two PCR amplicons, each of which was assumed to be amplified from a single locus, were classified into four types: markers generating polymorphic DNA fragments in all of the samples (codominant polymorphic markers, type I); markers generating polymorphic DNA fragments or no fragments (mixture of codominant and dominant polymorphic markers, type II); markers generating monomorphic DNA fragments in all of the samples (monomorphic markers, type III); and markers generating monomorphic DNA fragments or no fragments (dominant polymorphic markers, type IV). The numbers of types I, II, III, and IV markers were 16, 27, 17, and 17, respectively.

The average number of alleles per marker in the 43 codominant polymorphic markers (types I and II) was 3.6 alleles, ranging from 2 to 26 (Supplementary Table S2). Sixteen markers generated two alleles, while a single marker (CaES0089) generated 26 alleles. The average *HZ* value in the codominant markers (types I and II) was calculated as 0.30, ranging from 0.01 (CaES2489) to 0.89 (CaES0089) (Supplementary Table S2). Both the average number of alleles and the average *HZ* value were higher for type II markers (4.2 alleles/loci, *HZ* = 0.35) than for type I markers (2.8 alleles/loci, *HZ* = 0.21). Among the dominant markers (types II and IV), the average number of samples exhibiting the null allele was 17.1, ranging from just a single null allele for each of 14 markers to 167 null alleles for CaES4613 (Supplementary Table S2).

### Genetic distances and clustering of the 192 *Capsicum* lines

**Fig. 2:**
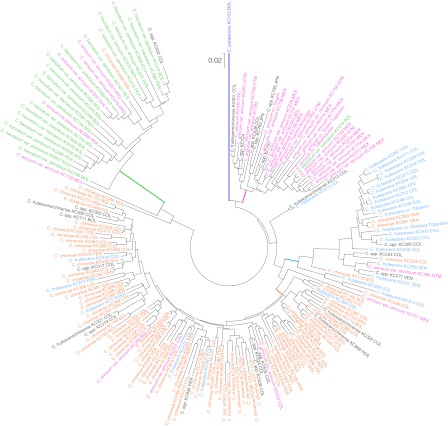
A *dendrogram* of *Capsicum* species based on genetic distances calculated by the neighbor-joining method. *C. annuum*, *C. baccatum*, *C. chinense*, *C. frutescens*, *C. pubescens*, and unclassified *Capsicum* spp. are shown in *red, green, orange, blue, purple*, and *black* *letters*, respectively

The genetic distances between all combinations of any two lines were investigated based on the genotyping data of the 60 informative markers (types I, II, and IV). The genetic distances among the 192 lines ranged from 0.00 to 0.39. A dendrogram was constructed, revealing 192 lines grouped into four clusters (Fig. 2). The four clusters correlated with species, with a few exceptions, and were designated Cluster A (*C. annuum*), Cluster B (*C. baccatum*), Cluster C (*C. chinense*), and Cluster F (*C. frutescens*) (Table 1; Supplementary Tables S1, S3). Cluster A consisted of 20 *C. annuum,* one *C. baccatum,* and six *Capsicum* spp. from the KIBR genetic resource center. Four *C. annuum* landraces, viz., Ougon, Fushimi-Amanaga, Shishi-Togarashi, and Takanotsume, also belonged to Cluster A. Cluster B comprised 20 *C. baccatum*, four *C. annuum*, one *C. chinense*, and one *Capsicum* spp. Cluster C, the largest cluster of the four, consisted of 78 *C. chinense*, seven *C. frutescens*, three *C. annuum*, and 13 *Capsicum* spp. Cluster F comprised 17 *C. frutescens*, five *C. chinense*, one *C. annuum*, three *Capsicum* spp., and two *C. furutescens* landraces, Okinawa-Togarashi and Tabasco. *C. pubescens* was located on the independent branch. Two *C. annuum* (KC139 and KC751), one *C. chinense* (KC262), one *C. furutescens* (KC515), and one *Capsicum* spp. (KC513) were not classifiable into any of the four clusters.

**Table 1:** The numbers of *Capsicum* species classified into each cluster based on the EST–SSR genotypes

Clusters	*C. annuum*	*C. baccatum*	*C. chinense*	*C. frutescens*	*C. pubescens*	*C.* spp.	Total
A	24	1	0	0	0	6	31
B	4	20	1	0	0	1	26
C	3	0	78	7	0	13	101
F	1	0	5	19	0	3	28
N	2	0	1	1	1	1	6
Total	34	21	85	27	1	24	192

### Sequence analysis of the chloroplast genes, *matK* and *rbcL*

DNA fragments were amplified from all 192 *Capsicum* lines with primers located within the two chloroplast genes, *matK* and *rbcL.* The sizes of the amplicons for *matK* and *rbcL* were 837 and 553 bp, respectively, excluding primer sequences.

In the *matK* sequencing analysis, two SNPs were found at the 129th (C/A) and the 312th positions (T/C) (Table 2; Supplementary Table S1). Of the 192 lines, 166 showed genotypes of C and T at the 129th and 312th positions, respectively. On the other hand, the remaining 26 samples exhibited A and C genotypes at the SNP sites. No other combinations of the SNPs were observed in the 192 samples. In the *rbcL*, on the other hand, one SNP (A/G) was found at the 392nd position (Table 2; Supplementary Table S1). Out of the 192 lines, 136 showed A on the SNP site, whereas the other 56 samples exhibited G.

**Table 2:** The numbers of *Capsicum* lines classified into each cluster based on the cytoplasmic haplotypes

Haplotypes		Clusters					Total
*matK*	*rbcL*	A	B	C	F	N	
CT	A	4	0	101	28	3	136
CT	G	27	0	0	0	3	30
AC	G	0	26	0	0	0	26

In total, three haplotypes were found in the plastid DNA of the 192 lines (Table 2). The haplotype CTA, that is, C and T at the 129th and the 312th positions of *matK*, respectively, and A in the *rbcL*, was found in Clusters C and F and four lines of Cluster A, viz., KC539, KC793, KC795, and Takanotsume, while the haplotypes CTG and ACG were predominantly specific to Clusters A and B, respectively.

### Identification of alleles specific to each cluster

By calculating the genotype frequencies of the 77 informative EST–SSRs, 19 genotypes were found to be specific to one of the four clusters (Table 3; Supplementary Table S4). Three genotypes of three markers were specific to Cluster A: a 102-bp fragment of CaES2655, a 166-bp fragment of CaES4192, and a 624-bp fragment of CaES5301 were found in 87, 97, and 90 % of the lines belonging to Cluster A, but were rarely seen in samples from the other clusters (between 0 and 4 %). On the other hand, 10 genotypes, of which one was the plastid DNA gene, *matK*, were detected in 89–100 % of the samples from Cluster B and were rarely found in samples classified as falling within the other clusters (between 0 and 6 %). In addition, two and four genotypes were specific to Clusters C and F, respectively.

**Table 3:** Frequencies of specific genotypes in the clusters

Markers	Genotypes (bp)	Cluster A (%)	Cluster B (%)	Cluster C	Cluster F
CaES2655	102	87	4	0	0
CaES4192	166	97	0	0	0
CaES5301	624	90	0	0	0
CaES0404	271	0	89	0	0
CaES1137	127	0	96	0	0
CaES2027	254	0	96	6	0
CaES2505	237	0	100	0	0
CaES2930	175	3	92	0	0
CaES4584	102	3	100	1	0
CaES4597	251	0	100	0	0
CaES4666	133/172	0	100	1	0
CaES5512	344	0	100	0	0
*matK*	AC	0	100	0	0
CaES1112	208	0	4	95	14
CaES4410	481	0	0	97	4
CaES2027	251	3	0	0	100
CaES2666	271	0	0	0	82
CaES4616	294	3	0	1	89
CaES4665	113	0	0	0	82

Predominant frequencies specific to the clusters are underlined

## Discussion

In the EST–SSR marker analysis, *Capsicum* lines from five species were classified into four clusters. The five species represented were *C. annuum* (Cluster A), *C. baccatum* (Cluster B), *C. chinense* (Cluster C), *C. frutescens* (Cluster F), and *C. pubescens*, which was represented by a branch rather than a cluster (Fig. 2). Although most of the samples could be classified according to species cluster, 22 samples did not fall into any obvious cluster (Table 1; Fig. 2). Based on the EST–SSR marker analysis, it was found that *C. chinense* and *C. frutescens* were closely related to *C. annuum*, and *C. baccatum* was distant from the other four species. This result confirmed previous reports based on isozyme, plastid DNA, and SNP analyses as well as morphological and cytogenetic analyses (Jarret 2008; Jeong et al. 2010; Walsh and Hoot 2001; and references therein).

The haplotypes of the plastid DNA of 22 samples fell into different clusters from those of their supposed species, as determined based on morphological traits (Supplementary Table S3). These haplotypes matched a dendrogram constructed from the EST–SSR marker sequences (Supplementary Table S1). This mismatch between the cluster and species name was also confirmed by AFLP analysis (Kim and Ban unpublished data). Two possibilities were considered for the mismatch between the classifications based on morphological traits and those based on DNA sequence. The first was misclassification of species based on morphological traits. Usually, classification of *Capsicum* species based on morphology is carried out by investigating characters of flowers, leaves, and fruits (IPGRI, AVRDC and CATIE 1995), but classification by this method is sometimes ambiguous. This is especially true for *C. chinense* and *C. frutescens*, since the morphological characteristics of the flowers are similar in these two species (Ishii and Ban unpublished data). Misclassifications between these two species are therefore more frequent than those between other species (Fig. 2; Table 1). Another possibility is genome introgression between different species. Because *Capsicum* can easily cross between species due to interspecific compatibilities, a small portion of alien genome might easily become fixed in both natural and field conditions. However, introgressed genomic regions seldom affect morphological traits, and only a few specific loci dramatically change plant phenotypes, e.g., plant height, number of fluorescent panicles, and fruit shape and size (Ashikari et al. 2005; Rodríguez et al. 2011). Therefore, such genomic introgressions would not be expected to result in changes in morphological characteristics that would lead to the observed mismatched classifications.

The euchromatic regions of the tomato genome were well represented by the CaES markers (Fig. 1), because analysis using a high-density genetic linkage map (Shirasawa et al. 2010) revealed that 1,792 EST–SSRs were in the gene-rich euchromatic regions (1 EST–SSR/130 kb), and 453 were in the gene-poor heterochromatic regions (1 EST–SSR/1,200 kb). In the family Solanaceae, comparative genomics have been advanced by using the conserved orthologous set II markers commonly mapped onto the linkage maps of different species (Wu et al. 2009; Wu and Tanksley 2010). Between pepper and tomato, comparative genomic study has revealed that the two species share 35 conserved synteny segments (Wu et al. 2009). Therefore, it might be possible to estimate the positions of the CaES markers on the *Capsicum* genome using the positions of the CaES markers on the tomato genome. This would greatly help the construction of high-density genetic linkage maps covering the whole genome of *Capsicum*. Alternatively, a combination of bin maps, using minimum sets of the marker loci to cover the genome generally, and fine maps, targeting specific loci using the CaES markers, would be useful.

The two “barcode” plastid genes, *rbcL* and *matK*, were insufficient to distinguish the tested *Capsicum* species. In the present study, the clusters C and F, which mainly consisted of *C. chinense* and *C. frutescens*, respectively, were not separated by the “barcode” sequences. Moreover, plastid DNA might not be suitable as a barcode in crops because of interspecific crossing. F1 hybrids from interspecific crossings are often used for cultivars due to their hybrid vigor, and plastid DNA cannot distinguish these hybrids from their maternal plants due to identical cytoplasm. Isogenic lines and introgression lines would also be indistinguishable from their maternal parents on the basis of plastid DNA. Our results indicated that four samples, viz., KC539, KC793, KC795, and Takanotsume, might be derived from such hybridizations between *C. annuum* as a paternal parent and either *C. chinense* or *C. frutescens* as a maternal parent because their nuclear and cytoplasmic genotypes belonged to Cluster A and Clusters C or F, respectively (Fig. 2; Supplementary Table S1). To overcome this problem, the intron sequence of the *waxy* gene encoded in the nuclear genome was proposed as a barcode (Jarret 2008; Walsh and Hoot 2001). However, the utility of *waxy* is strikingly limited because it has not been identified in all plant species. On the other hand, EST–SSR markers have also been useful for species identification (Table 3). A substantial amount of RNA sequence data has accumulated in public DNA databanks (DDBJ/EMBL/GenBank) since the production of transcribed sequence data is easily accomplished using next-generation sequencers. Advances in *in silico* searching of polymorphic SSR by comparative sequence data analysis (Shirasawa et al. 2012; polySSR: Tang et al. 2008; SSRpoly: http://acpfg.imb.uq.edu.au/ssrpoly.php) will accelerate the process of finding polymorphic SSR candidates. To correctly evaluate the genetic diversity of the samples in this study, polymorphic analysis of both nuclear and plastid genomes would be effective.

The CaES markers derived from EST sequences of *C. annuum* worked efficiently not only in *C. annuum* but also in *C. baccatum*, *C. chinense*, *C. frutescens*, and *C. pubescens*. In our previous study in tomato, 85 % of the EST–SSR markers derived from sequences of *S. lycopersicum* successfully amplified specific DNAs in a different species, *S. pennellii* (Shirasawa et al. 2010). In *Brassica*, the transferability of *B. rapa* EST–SSR markers between relatives was calculated as 43–100 %, depending on genetic distance (Ramchiary et al. 2011). In the case of *Capsicum* in this study, the transferability of the EST–SSR markers was 100 %, suggesting that nucleotide sequences in gene-coding regions of *Capsicum* species were substantially conserved.

Of the SSR motifs in the CaES markers, the most abundant motifs were poly (AAG)_n_ (17.0 %), poly (ATC)_n_ (11.3 %), poly (AAC)_n_ (10.1 %), and poly (AGC)_n_ (8.7 %) (Supplementary Fig. S1). This tendency almost matched that in tomato: poly (AAG)_n_ (22.5 %), poly (ATC)_n_ (12.1 %), poly (AGC)_n_ (9.3 %), and poly (AAC)_n_ (8.4 %) (Shirasawa et al. 2010). On the other hand, the abundant motifs in the EST–SSR markers in peanut were poly (AAG)_n_ (23.7 %), poly (AG)_n_ (19.8 %), poly (AAT)_n_ (8.2 %), and poly (GGT)_n_ (7.4 %) (Koilkonda et al. 2012), and those in radish were poly (AAG)_n_ (21.4 %), poly (GGA)_n_ (14.2 %), poly (ATC)_n_ (10.1 %), and poly (AAC)_n_ (8.0 %) (Shirasawa et al. 2011). While the prominent motif throughout the four species is poly (AAG)_n_, which is consistent with the previous report (Tóth et al. 2000), the distributions of the SSR motifs differed at the level of order but were similar to those of the family Solanaceae.

The present study used EST–SSR markers developed from publicly available EST sequences to reveal the relationships between *Capsicum* lines from the KIBR. Moreover, the positions of the markers on the tomato genome sequences were deduced. These markers and related information will contribute not only to species identification but also further QTL analysis, genome-wide association studies, and gene mapping towards the development of several attractive traits of *Capsicum*, e.g., fruit colors, shapes, sizes, and cellular components, in combination with morphological, biochemical, and histochemical methods.

## Electronic supplementary material

Below is the link to the electronic supplementary material.

Supplementary material 1 (PPT 119 kb)

Supplementary material 2 (XLS 53 kb)

Supplementary material 3 (XLS 1195 kb)

Supplementary material 4 (XLS 24 kb)

Supplementary material 5 (XLS 56 kb)
