# Supplementary material for: Development of Capsicum EST–SSR markers for species identification and in silico mapping onto the tomato genome sequence
Source: Mol Breed. 2012 Aug 11;31(1):101–10. doi: 10.1007/s11032-012-9774-z (PMC3538017; doi:10.1007/s11032-012-9774-z)
Supplement: Supplementary file 1 — Supplementary material 1 (PPT 119 kb) [file 11032_2012_9774_MOESM1_ESM.ppt]

## Slide 1
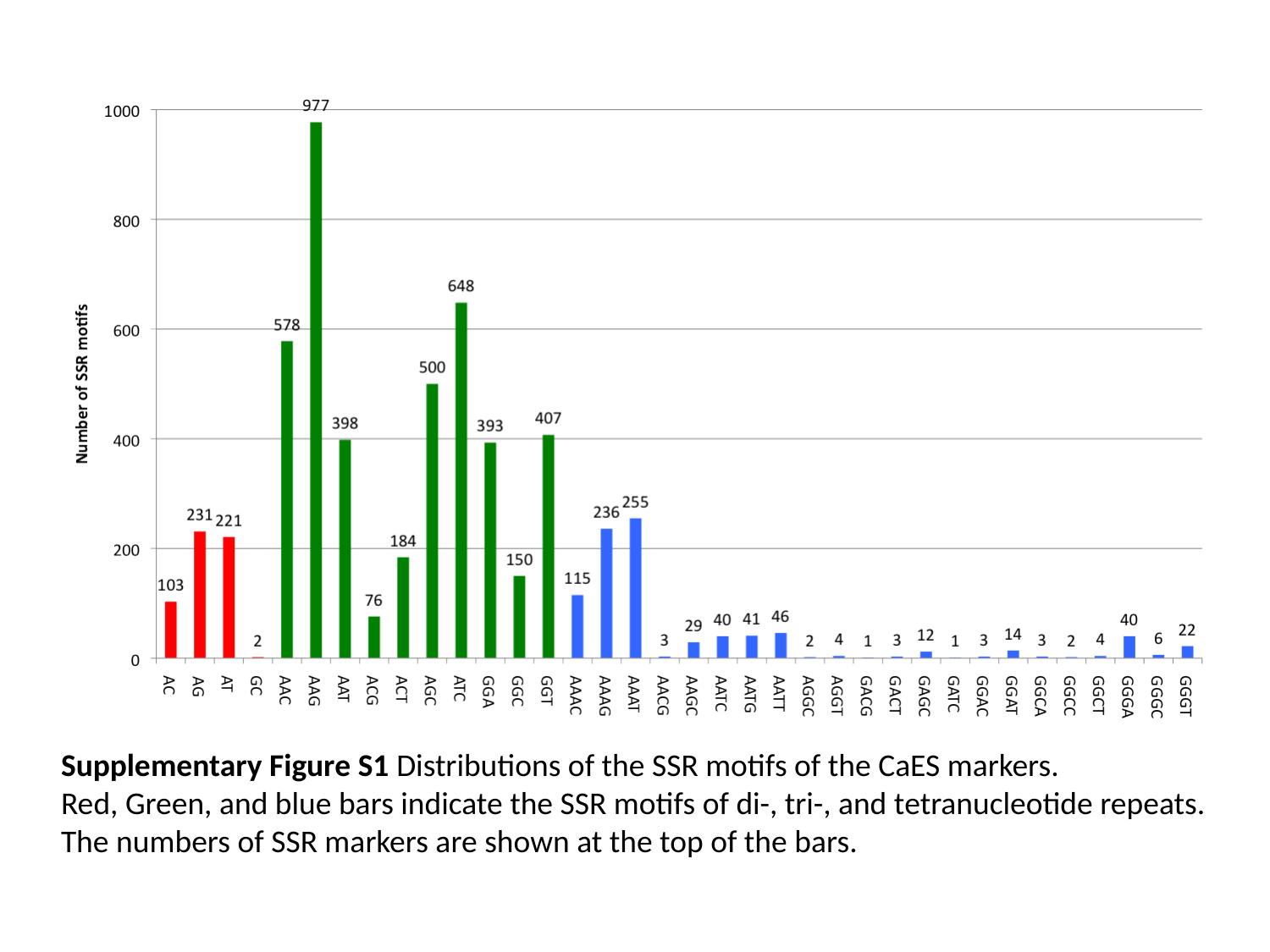

Supplementary Figure S1 Distributions of the SSR motifs of the CaES markers.
Red, Green, and blue bars indicate the SSR motifs of di-, tri-, and tetranucleotide repeats. The numbers of SSR markers are shown at the top of the bars.
